# Spleen Tyrosine Kinase Inhibition Mitigates Hemin-Induced Thromboinflammation in an Organ-Specific Manner in Sickle Cell Mice

**DOI:** 10.1161/ATVBAHA.124.322072

**Published:** 2025-08-15

**Authors:** Juma El-Awaisi, Gina Perrella, Nicolas Mayor, Veronika Tinkova, Simon J. Cleary, Beata Grygielska, Steve P. Watson, Jordan D. Dimitrov, Alexander Brill, Eman Hassan, Phillip L.R. Nicolson, Dean Kavanagh, Neena Kalia, Julie Rayes

**Affiliations:** Department of Cardiovascular Sciences, College of Medicine and Health, School of Medical Sciences, University of Birmingham, United Kingdom (J.E.-A., G.P., N.M., V.T., B.G., S.P.W., A.B., E.H., P.L.R.N., D.K., N.K., J.R.).; Faculty of Life Sciences & Medicine, School of Cancer & Pharmaceutical Sciences, Institute of Pharmaceutical Science, King’s College London, United Kingdom (S.J.C.).; Centre of Membrane Proteins and Receptors (COMPARE), Universities of Birmingham and Nottingham, The Midlands, United Kingdom (S.P.W.).; Centre de Recherche des Cordeliers (CRC), INSERM, Sorbonne Université, Université Paris Cité (USPC), Université Paris Descartes, Université Paris Diderot Paris, France (J.D.D.).

**Keywords:** hemin, hemoglobin, kidney, neutrophils, thromboinflammation

## Abstract

**BACKGROUND:**

Sickle cell disease (SCD) is a challenging genetic disorder characterized by hemolytic anemia, vaso-occlusive crises (VOC), and progressive organ damage. Despite its severity, effective treatments are limited. The recent withdrawal of promising therapies, such as the anti–P-selectin antibody Crizanlizumab and the hemoglobin polymerization inhibitor Voxelotor, highlights the urgent need for innovative approaches to alleviate vaso-occlusion and thromboinflammation.

**METHODS:**

In this study, we used advanced techniques, including intravital microscopy, laser speckle contrast imaging, and histological analysis, to examine the role of syk (spleen tyrosine kinase) in platelet and neutrophil recruitment, and blood perfusion in the lung, kidney, liver, and spleen of SCD mice.

**RESULTS:**

In the Berkeley SCD model, hemin-induced vaso-occlusion and impairment in pulmonary blood perfusion were independent of red cell congestion and fibrin deposition. Hypoperfusion was driven by adhesion of neutrophils and platelets in the microcirculation and exacerbated by pulmonary emboli. Hemin-induced cell adhesion and hypoperfusion were also observed in the renal microcirculation, whereas it was limited in the liver and spleen of SCD mice, suggesting that organ-specific mechanisms drive hypoperfusion and vaso-occlusion. To explore therapeutic options, we investigated the potential of Syk inhibition in improving blood perfusion and reducing thrombo-inflammation in different organs. Selective Syk inhibition, using BI-1002494, reduced cellular adhesion in the pulmonary and renal microvasculature, effectively restoring blood perfusion and reducing thrombo-inflammation. Low-dose Syk inhibitor was effective in reducing neutrophil adhesion and improving blood perfusion without inducing bleeding. Increasing the dose exacerbated hemin-induced bleeding in the lungs, likely due to off-target activity againt other kinases, including Src.

**CONCLUSIONS:**

These findings underscore the critical role of Syk in platelet and neutrophil mediated-thrombo-inflammation and hypoperfusion in SCD, suggesting that Syk inhibition is a promising strategy to reduce organ-specific vaso-occlusion, improve renal and pulmonary perfusion, and reduce organ damage.

**Highlights**

- Hemin-induced organ-specific mechanisms of thrombo-inflammation and vaso-occlusion in sickle cell mice.
- Hemin increases neutrophil adhesion on fibrinogen and VWF (von Willebrand Factor) in a Syk (spleen tyrosine kinase)-dependent manner.
- Inhibition of Syk using BI-1002494 reduces neutrophil and platelet recruitment to the lung and kidney and improves organ perfusion.

Sickle cell disease (SCD) is an inherited severe hemoglobinopathy caused by a mutation in the beta globin gene *HBB* leading to hemoglobin S (HbS), which upon deoxygenation induces red blood cell (RBC) sickling, adhesion, and lysis.^1^ This results in chronic hemolytic anemia, vaso-occlusive crises (VOC), impairment in organ perfusion, and progressive organ damage.^2,3^ Affected individuals suffer an elevated risk of developing acute chest syndrome (ACS), a potentially fatal complication associated with high levels of inflammatory markers.^4,5^ Hydroxyurea, which increases fetal hemoglobin, reduces the frequency of painful crises and infections,^6,7^ however, neutrophils, key drivers of lung injury,^8,9^ remain active in hydroxyurea-treated patients.^10^ Recent Food and Drug Administration approved CRISPR-Cas9 (clustered regularly interspaced short palindromic repeats/clustered regularly interspaced short palindromic repeat–associated 9) therapies aiming to increase fetal hemoglobin (Exagamglogene autotemcel, Casgevy) or the production of modified hemoglobin A (HbA^T87Q^; Lyfgenia) have shown promising results in reducing VOC^11–14^; however, long-term efficacy and impact on organ decline and life expectancy are still under investigation. Additionally, recent withdrawals of crizanlizumab and voxelotor from the market have left patients in need of alternative therapies.^15–17^

Besides physical obstruction, RBC lysis releases hemoglobin gene and heme, both of which provoke VOC and ACS.^18,19^ Once released in the extracellular space, heme acts as a potent prothrombotic and proinflammatory damage-associated molecular pattern, driving thrombosis and inflammation in various organs.^20^ Free oxidized heme (hemin) activates endothelial cells through TLR-4 (toll-like receptor 4),^18^ induces NET (neutrophil extracellular trap) formation,^21,22^ and platelet activation through CLEC-2 (C-type lectin receptor 2) and GP-VI (glycoprotein VI),^23–25^ all of which are regulated by Syk (spleen tyrosine kinase).

Evidence suggests that inhibiting Syk in SCD mice may improve hypoperfusion and reduce VOC. First, heme activation of CLEC-2 and GPVI leads to Syk phosphorylation and platelet activation.^26^ Second, Syk is essential for neutrophil adhesion^27^ and their recruitment to the lung vasculature.^8^ Third, the engagement of PSGL-1 (P-selectin glycoprotein ligand 1) by P-selectin and E-selectin transactivates Syk in leukocytes, reducing neutrophil rolling velocities.^28–30^ Moreover, neutrophil interaction with immobilized P-selectin activates Syk.^31^ Fourth, Syk phosphorylation is elevated in deoxygenated sickle RBCs^32^ and Syk inhibition ex vivo blocks band 3 tyrosine phosphorylation inhibiting RBC adhesion and deformability in flow models.^33^ Fifth, Syk is involved in NLRP3 (NLR family pyrin domain containing 3) inflammasome activation and IL-1β (interleukin 1β) secretion,^34^ and Syk inhibitor R406 reduced platelet inflammasome NLRP3 activation and thrombus formation ex vivo.^35^ The safety and efficacy of Syk inhibitors in immune thrombocytopenia suggest potential for SCD; however, the in vivo impact on organ perfusion and vaso-occlusion remains to be determined.^36–38^

In this study, we assessed the effect of the experimental Syk inhibitor BI (Boehringer Ingelheim)-1002494 on platelet and neutrophil recruitment to the lungs, liver, spleen, and kidney in SCD mice, both at baseline and following hemin treatment. We also evaluated its impact on vascular integrity and organ perfusion.

## Methods

The data that support the findings of this study are available from the corresponding author on reasonable request.

### Human Blood

Venous blood was drawn from healthy, consenting, drug-free volunteers (males and females) into 3.2% trisodium citrate BD Vacutainers (Becton Dickinson, United Kingdom). Ethical approval was granted by the University of Birmingham Research Ethics Committee (ERN_11-0175), and blood collection followed ethical principles outlined in the Declaration of Helsinki.

### Mice

Berkeley SCD mice^39^ were purchased from Jackson Laboratories. Age-matched males and females sickle (*Hba*^0/0^ *Hbb*^0/0^ Tg(Hu-miniLCRα1^G^γ^A^γδβ^S^) and non-sickle control mice (*Hba*^0/0^ *Hbb*^0/+^ Tg(Hu-miniLCRα1^G^γ^A^γδβ^S^; 8–14 weeks) on EURodent diet 14% (5LF2; Labdiet) were used. Sickle mice have a genetic defect due to a mutation on the beta globin gene, and the number of sickle mice generated is relatively low to perform these studies in males and females. As the data show no difference in the response due to the acute nature of this model (1 hour), data from each sex were combined. All experiments complied with UK law (Animals Scientific Procedures Act 1986) and received approval from the local ethics committee and the UK Home Office under PPL PP5212425 granted to the University of Birmingham.

### Intravital Imaging of Hemin-Induced Thrombo-Inflammation and Perfusion in Ventilated Lungs

Mice were terminally anesthetized with intraperitoneal ketamine (100 mg/kg) and medetomidine (10 mg/kg) injections, intubated, and ventilated with oxygen (stroke volume: 220 μL, respiratory rate: 130 breaths/min; minivent rodent ventilator, Biochrom Ltd/Harvard Apparatus). Vaso-occlusion episodes (VOE) was induced by intravenous injection of 20 µmol/kg of hemin (Frontiers specialty chemicals). Hemin was prepared in 50 mmol/L NaOH, diluted in PBS, pH adjusted to 7.3, and filtered before injection. EDTA-anticoagulated blood was collected through retro-orbital sampling for cell count analysis (X Pentra 80, HORIBA). Syk inhibitor BI-1002494 (4 or 20 mg/kg; Boehringer Ingelheim, Germany) or vehicle (PBS-10% DMSO [dimethyl sulfoxide]) was injected intraperitoneally 30 minutes before hemin. Real-time intravital observations were performed as previously described.^40,41^ Briefly, a thoracic window (steel stabilizer) was used to apply gentle negative pressure (≈20 mm Hg) to a region of the surface of the upper part of the left lobe of the lung, allowing stabilization with maintained ventilation^41^ (Figure S1A). To simultaneously image endogenous myeloid cells and platelets, PE (phycoerythrin) anti–mouse Gr 1 (granulocyte-receptor 1 antigen; Ly-6G/Ly-6C; clone RB6-8C5; BioLegend) and DyLight 649 anti- GP (glycoprotein) Ibβ (GPIb-beta; X-649, Emfret) were injected 5 minutes before imaging. Alexa-488 anti-mouse Ly6G antibody (Clone 1A8; BioLegend) was used to confirm neutrophil recruitment (Figure S1B). In contrast, visualizing deeper lobes, such as the right caudal and accessory, was challenging using this cellular-level imaging technique (Figure S1C). Intravital imaging was performed using an upright microscope (BX61WI; Olympus) equipped with a Nipkow spinning disk confocal head (Yokogawa CSU) and an Evolve EMCCD camera (Photometrics).^42^ The first 1-minute capture was performed at baseline followed by hemin injection. Images were then captured every 15 minutes in the same area for 1 hour at both ×10 and ×40 magnification. At the end of the imaging period, the stabilizer was moved to the left kidney, spleen, and liver for additional images. Mice were culled by cervical dislocation, and organs were collected for histology. Data were captured, stored, and analyzed using Slidebook 6 software (Intelligent Imaging Innovations). Neutrophils and platelet aggregates/microthrombi at ×10 magnification were quantified by using segment masks on PE-Gr-1 and DyLight 649 anti-GPIbβ staining, respectively. Integrated fluorescence density, accounting for size and fluorescence intensity, was calculated using ImageJ.

### Laser Speckle Contrast Imaging of the Ventilated Mouse Lungs

Laser speckle contrast imaging quantified left pulmonary blood flow in mice as previously described.^43^ Following the surgical preparation described earlier, the laser speckle contrast imaging device (moorFLPI-2; Moor Instruments, United Kingdom) was positioned above the exposed left lobe. A defined area for flux data collection was marked at baseline and 1-hour post-hemin treatment. Additional recordings of the left kidney, spleen, and liver were taken both at baseline and 1-hour post-hemin treatment. Using the manufacturer-supplied image software (mFLPI2Measure V2.0; mFLPIReview V5.0), 1000 frames were captured at each time point with a frame rate of 25 Hz and spatial processing (sliding window, time constant: 0.1 seconds). In-house-developed basic speckle analysis software (SpAn; open source, available online at https://github.com/kavanagh21/SpAN) facilitated the identification and compilation of flux values.

### Histology

Lung, kidney, spleen, and liver were collected from unchallenged control and SCD mice and from mice following intravital imaging procedures. Lung and liver lobes from all mice were processed for histological analysis. To minimize potential artifacts or damage, particularly bleeding, caused by the application of the stabilizer, we examined bleeding only in lobes that had not been stabilized. Accordingly, the left lung lobe was used for intravital imaging, whereas the right lung lobes were reserved for histology to assess bleeding. Organs were immediately fixed in 4% paraformaldehyde overnight, dehydrated, and embedded in paraffin. Formalin-fixed paraffin-embedded sections (6 µm) were stained with hematoxylin and eosin (Abcam). Organs from untreated non-sickle and SCD mice served as baseline controls. Formalin-fixed paraffin-embedded sections were processed for immunofluorescence and stained for mouse fibrinogen/fibrin (YNGMFBG7S; Accurate Chemical and Scientific Corporation) and S100A9 (AF2065; R&D), followed by secondary donkey anti-goat Alexa-647 antibody. Nuclei were counterstained with Hoechst 33342 (Sigma-Aldrich). Sections were mounted with ProLong Gold Antifade Mountant (Thermo Fisher Scientific); images were captured and analyzed using a Zeiss Axio Scan.Z1 microscope and ZEN software.

### Human Platelet Aggregation

Human washed platelets were prepared, and aggregation was performed as previously described.^24^ Platelets were preincubated with BI-1002494 or PRT-060318 (1 or 10 µmol/L) for 15 minutes before stimulation with hemin (5 µmol/L). Aggregation was assessed using a PAP-8E aggregometer in the presence of CaCl_2_ (2 mmol/L).

### Western Blot

Human washed platelets (2×10^8^/mL) containing 2 mmol/L CaCl_2_ were pretreated with eptifibatide (integrilin, 9 µmol/L) before stimulation with hemin (5 µmol/L) or CRP (collagen-related peptide, 3 µg/mL) under stirring conditions. Platelets were pretreated with DMSO (vehicle) or BI-1002494 (1 or 10 µmol/L) for 15 minutes at 37 °C before hemin addition. Platelet samples were collected before stimulation with hemin or CRP under stirring conditions (1200 rpm) and after 1- and 6-minute stimulation into Laemmli sample buffer. Platelet lysates were separated by SDS-PAGE (4%–12%) under reduced conditions and immunoblotted using rabbit anti-pSrc (Tyr418; 1:500, Cell Signaling Technology), and rabbit anti-LAT Y191 (Upstate Biotechnology Inc). The membranes were stripped, blocked, and re-probed with anti-GAPDH antibody (Cell Signaling Technology) used as the loading control. Uncropped gel images are shown in the Supplemental Material. Images were acquired using LICOR Odyssey imager, and band quantification was measured using Image Studio.

### Neutrophil Adhesion and NETosis

Neutrophils were isolated from EDTA-anticoagulated blood using Histopaque 1077 and 1119 (Sigma-Aldrich). For adhesion assays, fibrinogen (200 µg/mL) and VWF (von Willebrand Factor; 100 µg/mL; Wilfactin, LFB, France) were coated on 24-well plates for 1 hour at 37 °C, blocked with 7.5% BSA for 1 hour, and washed with PBS. Purified neutrophils (0.5 million per well) were added and incubated for 30 minutes at 37 °C. TNF (tumor necrosis factor)-α (10 ng/mL; Peprotech) and hemin (5 µmol/L) were then applied for 30 minutes at 37 °C. In some conditions, neutrophils were preincubated with BI-1002494 or PRT-060318 (10 µmol/L) for 15 minutes before TNF-α and hemin addition. Following incubation, cells were washed, fixed and adhesion quantified using EVOS microscopy. The average neutrophil count from 5 representative images per donor was analyzed.

For NETosis experiments, neutrophils (200 000 per well) were added on poly l-Lysine for 30 minutes before stimulation with phorbol myristate acetate PMA (100 nmol/L) or uric acid (20 µg/mL) for 3 hours. For experiments in the presence of platelets, human washed platelets (20 million) were stimulated with hemin (5 µmol/L) for 20 minutes before addition to neutrophils. Unstimulated platelets were used as controls. In some conditions, platelets were pretreated with BI-1002494 (10 µmol/L) before the addition of hemin. In some conditions, hemin-activated platelets were added to neutrophils pretreated with BI-1002494. Following a 3-hour incubation at 37 **°**C, cells were washed, fixed, and stained with Sytox green for 5 minutes and DNA stain imaged using EVOS microscopy. DNA area coverage of 5 separate fields/condition was measured using ImageJ.

### VWF and S100A8/A9 Elisa

Plasma levels of VWF were quantified by ELISA using rabbit anti-VWF antibody (Dako) and HRP-conjugated anti-VWF antibody (Dako), with plasma-derived human VWF as the standard. S100A8/A9 levels were measured using the mouse S100A8/A100A9 heterodimer DuoSet ELISA (R&D).

### Statistical Analysis

Data are presented as mean±SD, unless otherwise specified. Statistical significance between 2 groups was determined using an unpaired Student *t* test, whereas differences among multiple groups were assessed using 1-way ANOVA with Tukey or Dunnett multiple comparisons test, or the Kruskal-Wallis test, as indicated in the figure legends. Analyses were performed using Prism 10 (GraphPad Software, Inc).

## Results

### Hemin Administration Caused a Sharp Drop in Lung Perfusion in SCD Mice Independent of Fibrin Deposition and Red Cell Congestion

A dose of 75 µmol/kg of hemin induced 100% lethality in SCD mice, whereas a lower dose (17.5 µmol/kg) did not induce lethality within 2 hours of treatment.^19^ Based on these findings, we selected a 20 µmol/kg dose to investigate the organ-specific effects of hemin on platelet and myeloid cell recruitment, as well as organ perfusion.

**Figure 1.**
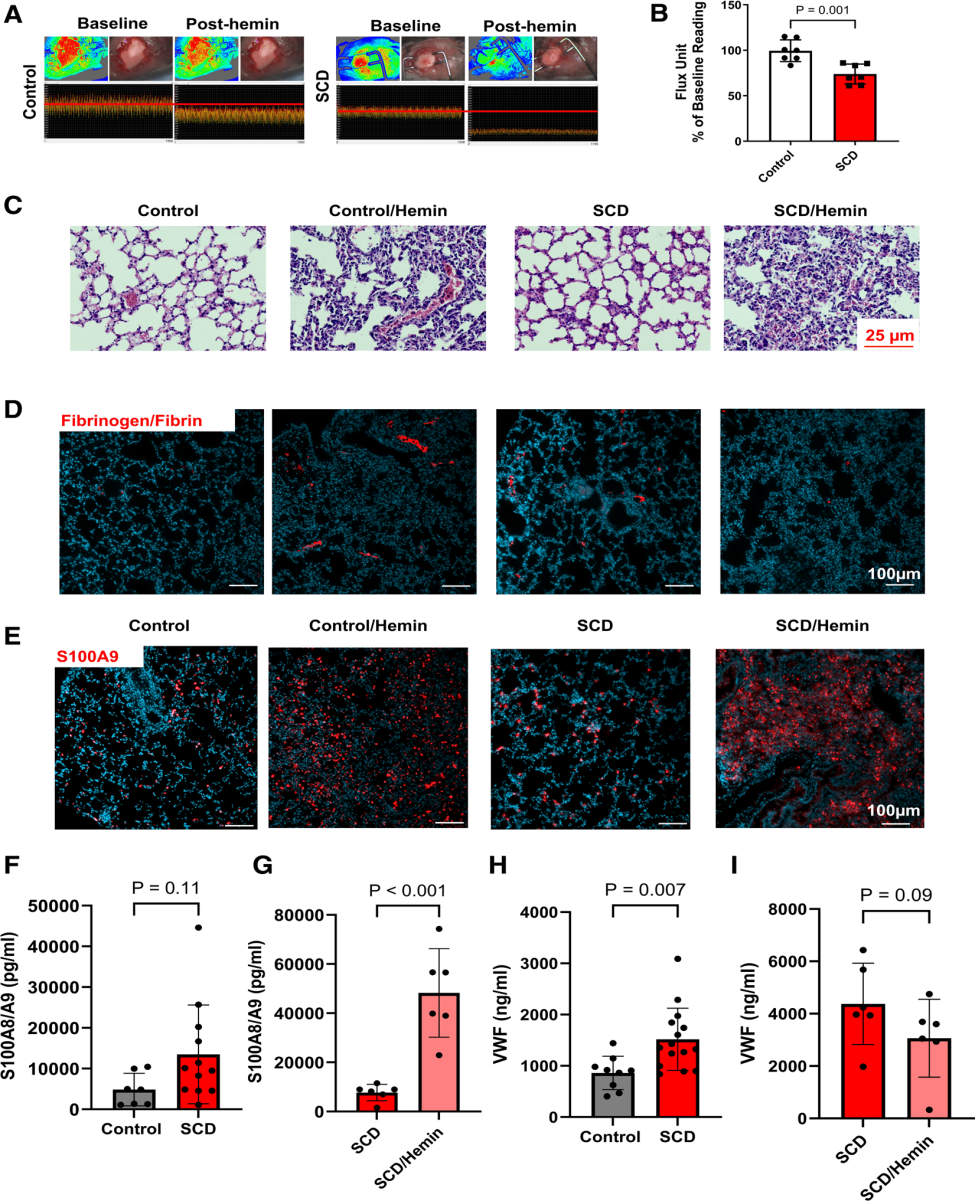
**Hemin impairs lung perfusion in ventilated sickle cell disease (SCD) independent of coagulation and red cell congestion.** Hemin (20 µmol/kg, IV) was injected in ventilated control and SCD mice. **A**, Representative laser speckle contrast imaging (LSCI) images showing flux data, flux heat maps, and a corresponding photo of the ventilated lungs. **B**, Quantitative analysis of flux unit readings as a percentage of baseline values obtained by LSCI (n=7 mice). **C**, Hematoxylin and eosin (H&E) staining of formalin-fixed paraffin-embedded (FFPE) lung sections in untreated and hemin-treated mice. Scale bar 25 µm. **D** and **E**, Immunofluorescence staining for (**D**) fibrinogen/fibrin and (**E**) S100A9 in FFPE sections. Nuclei are stained with Hoechst 33342 (blue). Scale bar 100 µm. H&E and immunofluorescence staining were imaged using a Zeiss Axio Scan microscope and analyzed using Zen software (n=3 per group). **F** through **I**, Plasma from control and SCD mice was collected at baseline and 1 hour prior to hemin injection. Plasma levels of S100A8/A9 and VWF (von Willebrand Factor) were measured by ELISA. The statistical significance between 2 groups was analyzed using an unpaired *t* test for **B**, **F**, and **G** and a paired *t* test for **G** and **I**.

At baseline, SCD mice exhibited lower RBC and platelet counts, whereas white blood cell and neutrophil counts were elevated compared with controls (Figure S2A through S2D). Lung and kidney perfusion were similar in SCD and control mice, but liver and spleen perfusion were significantly reduced in SCD mice (Figure S2E through S2H). Hemin injection did not affect RBC, platelet, or total white blood cell counts in control mice, but neutrophil counts increased significantly pos-themin treatment (Figure S3A through S3D). In contrast, hemin reduced RBC, platelet, white blood cell, and neutrophil counts in SCD mice (Figure S3E through S3H). Hemin induced a sharp decline in pulmonary blood perfusion in SCD mice compared with controls, measured 1 hour post-injection (Figure 1A and 1B). Histological analysis revealed local bleeding in all lobes, with no obvious red cell congestion in the ventilated lung (Figure 1C).

Hemin was shown to rapidly activate coagulation in mice. We investigated whether blood perfusion impairment was linked to fibrin deposition in the lung microcirculation. Immunofluorescence analysis of formalin-fixed paraffin-embedded lung sections collected 1 hour after hemin treatment revealed fibrin in large vessels of control mice but not in SCD mice treated with hemin (Figure 1D), indicating that pulmonary hypoperfusion is not due to coagulation activation or fibrin-induced vessel occlusion. Following hemin injection, there was rapid recruitment of S100A9-positive cells, predominantly neutrophils, to the lungs of both control and SCD mice (Figure 1E). Although S100A8/A9 plasma levels were not significantly different in SCD and control mice at baseline (Figure 1F), hemin significantly increased these levels, suggesting neutrophil activation (Figure 1G). Although VWF levels were elevated in SCD mice at baseline, hemin did not further increase these levels (Figure 1H and 1I). These findings suggest that hemin-induced lung hypoperfusion is linked to immune cell recruitment and activation, primarily neutrophils, rather than fibrin deposition or coagulation activation.

### Syk-Dependent Neutrophil Adhesion in the Pulmonary Microcirculation Supported Lung Hypoperfusion in Response to Hemin

Platelets and neutrophils cooperate to induce VOC and ACS in SCD.^8,9,44^ Syk phosphorylation mediates platelet activation by hemin,^24^ neutrophil adhesion,^30^ NET formation,^45^ and P-selectin-dependent neutrophil recruitment.^31^

**Figure 2.**
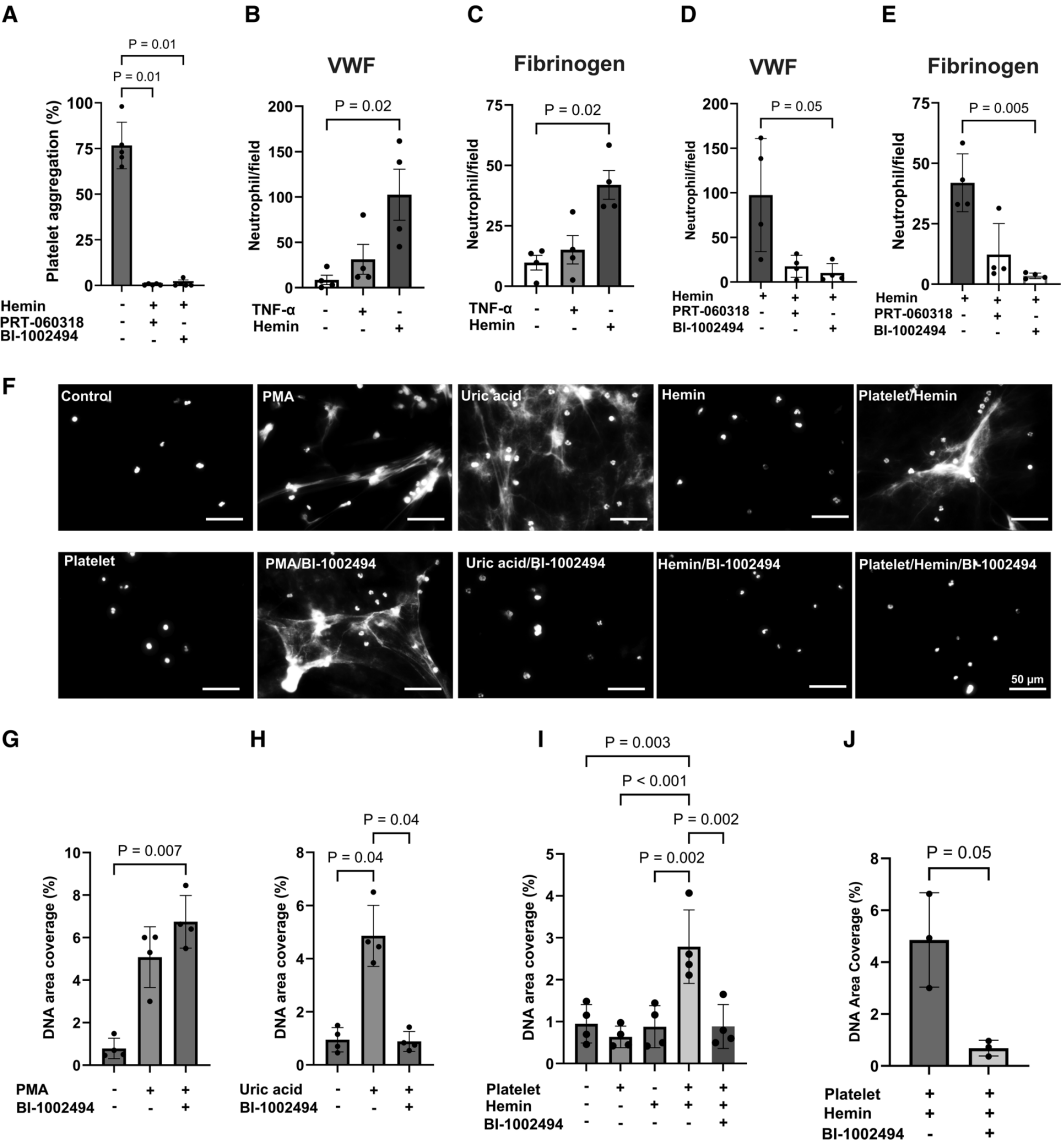
**Syk (spleen tyrosine kinase) inhibitors BI-1002494 and PRT-060318 reduced neutrophil adhesion, NETosis, and platelet aggregation by hemin. A**, Human washed platelet aggregation by hemin (5 µmol/L) was assessed by light transmission aggregometry. Platelets were pretreated for 10 minutes at 37 °C with PRT-060318 (10 µmol/L) before stimulation with hemin. **B** and **C**, Purified neutrophil adhesion (30 minutes at 37 °C) on (**B**) immobilized VWF (von Willebrand Factor; 100 µg/mL) or (**C**) fibrinogen (200 µg/mL) was assessed in the presence or absence of TNF-α (tumor necrosis factor α; 10 ng/mL) or hemin (5 µmol/L; n=4 independent experiments, average of 5 images). **D** and **E**, Neutrophils were pretreated with Syk inhibitors, PRT-060318 or BI (Boehringer Ingelheim)-1002494 (10 µmol/L), before incubation with hemin on (**D**) VWF or (**E**) fibrinogen (**B** and **D** were performed in the same experimental settings and the data points in hemin-treated group in **B** were used as control for **D**; some data points in hemin-treated group were used in **C** and **E**). **F** through **J**, Neutrophil extracellular trap formation in vitro. **F**, Representative image of DNA stain using Sytox Green. Scale bar 50 µm. **G** and **H**, Neutrophils were treated with PMA (phorbol 12-myristate 13-acetate; 100 nmol/L), uric acid (20 µg/mL). **I**, Nonactivated or hemin-activated platelets were added to neutrophils for 3 hours. Platelets were pretreated with BI-1002494 (10 µmol/L) before hemin stimulation. **J**, Hemin-activated platelets were added to neutrophils pretreated with vehicle or BI-1002494 for 3 hours. **G** through **J**, Area coverage of DNA was quantified and averaged from 5 separate fields per condition in each donor, and the results shown as mean±SD of 3 to 4 independent donors and experiments. The significant difference between multiple groups was assessed using Kruskal-Wallis multiple comparison test with post hoc Dunn analysis compared with hemin (**A**), unstimulated neutrophils (**B**, **C**, and **G**), hemin-stimulated neutrophils (**D** and **E**), uric acid (**H**), platelet/hemin (**I**). For **J**, paired *t* test was performed.

To evaluate the role of Syk in neutrophil and platelet activation by hemin, we tested the efficacy of 2 selective Syk inhibitors, BI-1002494 and PRT-060318, on hemin-induced neutrophil adhesion, NETosis, and platelet aggregation in vitro using neutrophils and platelets isolated from healthy donor blood. In line with our previous study,^24^ both inhibitors (10 µmol/L) completely abolished hemin-induced platelet aggregation as assessed by light transmission aggregometry (Figure 2A). Hemin-mediated platelet aggregation was also inhibited by lower doses of BI-1002494 (1 µmol/L; Figure S4A). BI-1002494 used at 1 and 10 µmol/L inhibited platelet aggregation and LAT (linker for activation of T cells) Y191 phosphorylation confirming the efficacy of both doses in inhibiting platelet activation by hemin. However, at 10 µmol/L, BI-1002494 inhibited also Src Y418, suggesting an off-target activity against Src (Figure S4B through S4D; raw gels in Supplemental Material). Therefore, at high concentration, BI-1002494 inhibits both Syk and Src, whereas at lower concentrations, a more selective effect on Syk is observed. Hemin (5 µmol/L) significantly increased neutrophil adhesion to VWF and fibrinogen, although TNF-α had no effect (Figure 2B and 2C). Both BI-1002494 and PRT-060318 inhibited hemin-induced neutrophil adhesion to VWF and fibrinogen (Figure 2D and 2E). Hemin did not induce NET formation in healthy neutrophils, but the presence of platelets significantly enhanced NETosis (Figure 2F and 2I). We further focused on BI-1002494 to confirm its efficacy in blocking NETosis. PMA, a Syk-independent inducer of NETosis, showed no change with BI-1002494 treatment; however, uric acid-induced NETosis, a Syk-dependent process, was significantly inhibited, confirming BI-1002494 effect on Syk-dependent NETosis (Figure 2F through 2H). Additionally, NETosis driven by activated platelets was inhibited when platelets were pretreated with BI-1002494 (Figure 2F and 2I). Pretreating neutrophils with BI-1002494 before exposure to hemin-activated platelets also inhibited NET formation (Figure 2J). Thus, Syk inhibition blocked neutrophil adhesion, platelet activation, and Syk-dependent NETosis induced by hemin.

**Figure 3.**
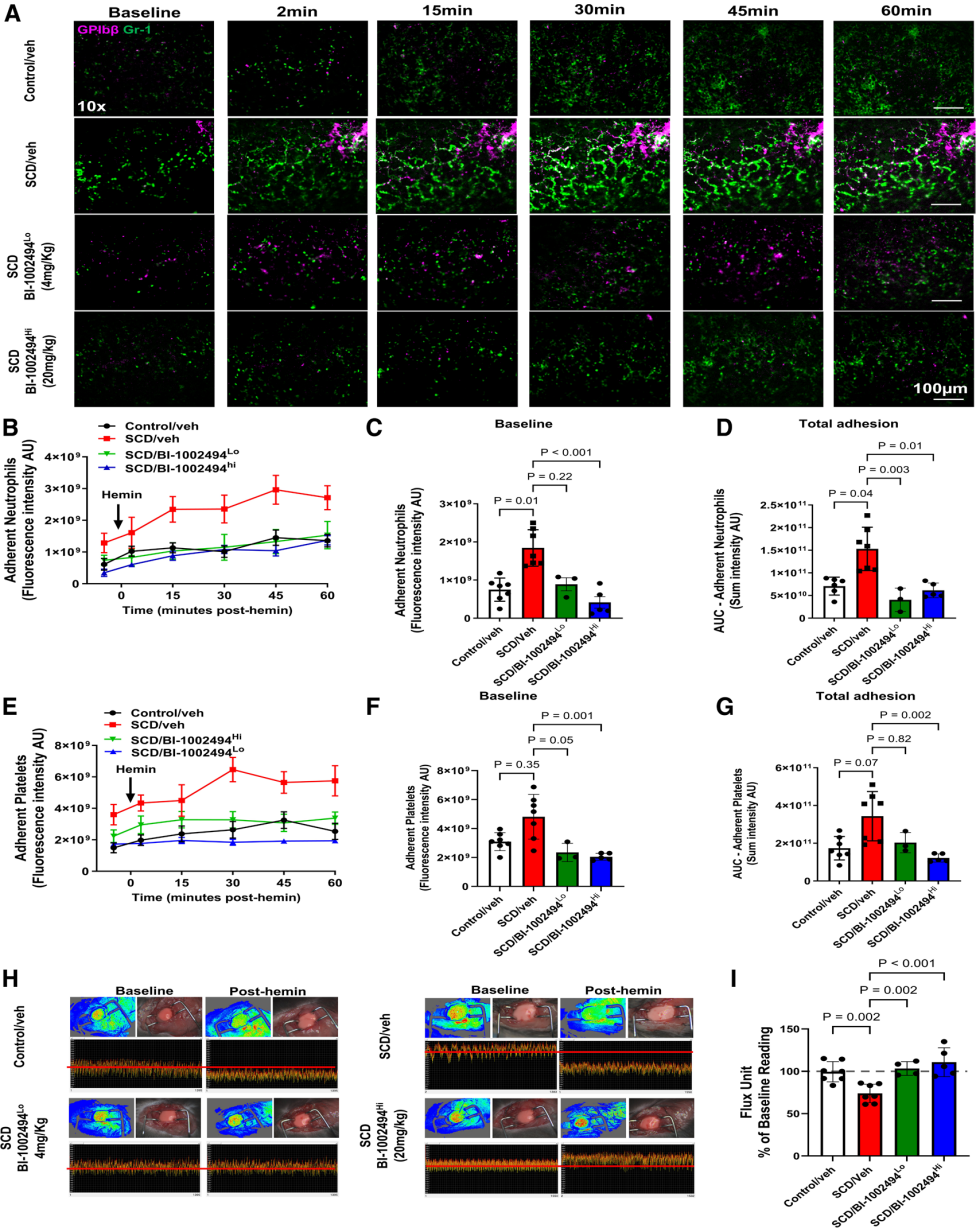
**Syk (spleen tyrosine kinase) inhibitor BI-1002494 impairs hemin-induced neutrophil and platelet recruitment to lungs and reverses hypoperfusion.** BI-1002494 (4 or 20 mg/kg) or vehicle (veh) were injected in sickle cell disease (SCD) mice via intraperitoneal route 30 minutes before the injection of hemin (20 µmol/kg, IV). **A**, Representative intravital images of the ventilated lungs showing adherent neutrophils (Gr [granulocyte-receptor antigen]-1; green) and platelets (GP [glycoprotein] Ibβ; magenta) in the pulmonary vasculature over a time course of 60 minutes at ×10 magnification. Scale bar 100 µm. Quantitative analysis of the intravital data for adherent neutrophils (**B**) over the time course as fluorescence intensity arbitrary unit (AU), at (**C**) baseline, (**D**) and the area under the curve (AUC) analysis over a time course of 60 minutes. Quantitative analysis of the intravital data for platelets (**E**) over the time course as fluorescence intensity AU, at (**F**) baseline, (**G**) and AUC analysis over a time course of 60 minutes. **H**, Representative laser speckle contrast imaging (LSCI) images showing flux data, flux heat maps, and a corresponding photo of the ventilated lungs. **I**, Quantitative analysis of flux unit readings as a percentage of baseline values obtained by LSCI. The statistical significance between multiple groups was analyzed using Kruskal-Wallis multiple comparison test with post hoc Dunn analysis compared with SCD/veh group for **C**, **D**, **F**, **G** and ordinary 1-way ANOVA with Holm-Šídák multiple comparisons test for **I** (n=3–7).

We next evaluated the kinetics of platelet and neutrophil recruitment to the pulmonary microcirculation using intravital imaging, assessing whether the Syk inhibitor BI-1002494 affects cell recruitment and hypoperfusion compared with vehicle-treated mice. Pretreatment with vehicle, followed by hemin injection, did not affect platelet or neutrophil recruitment to the lung microcirculation of control and SCD mice in comparison to untreated control and SCD mice (Figure S5A through S5D); thus, vehicle treatment was used as the control in subsequent experiments. Intravital imaging revealed significantly higher neutrophil recruitment to the pulmonary microcirculation at baseline and after hemin injection in SCD mice compared with controls (Figure 3A through 3D). Dual labeling of myeloid cells (Ly6G/Ly6C, clone RB6-8C5) and neutrophils (Ly6G, clone 1A8) showed that nearly all recruited cells were neutrophils (Figure S1B and S1C). Similarly, Ly6G-positive cells were recruited to the right cranial and medial lobes (Figure S1B and S1C). Therefore, it is likely that neutrophil recruitment was observed across multiple lung lobes, reflecting a systemic response as assessed by S100A9 and Ly6G staining (Figure 1E; Figure S1B and S1C). In contrast to the stable adhesion of neutrophils, platelet adhesion, both stable and transient single-platelet adhesion as well as aggregate formation, was delayed relative to neutrophils (Figure 3E through 3G; Videos S1 and S2). Large platelet emboli were observed in the pulmonary microcirculation of hemin-treated SCD mice (Video S2). The increased neutrophil recruitment, along with delayed platelet adhesion and emboli formation, was associated with impaired lung perfusion, as measured by laser speckle contrast imaging (Figure 3H and 3I).

Previously, prophylactic or therapeutic administration of BI-1002494 (100 mg/kg) by oral gavage was shown to protect mice against arterial thrombosis and reduce brain infarct in an ischemic stroke model, without affecting hemostasis.^46^ In the present study, 2 doses of BI-1002494 (20 and 4 mg/kg) were administered via intraperitoneal injection 30 minutes before hemin administration. At low dose, BI-1002494 treatment did not affect basal lung perfusion in SCD mice (Figure S6). The higher dose (20 mg/kg, BI-1002494^Hi^) inhibited both baseline and hemin-induced platelet and neutrophil recruitment to the lung, improving perfusion following hemin administration in SCD mice (Figure 3). The lower dose (4 mg/kg, BI-1002494^Lo^) reduced neutrophil adhesion and, to a lesser extent, platelet adhesion, with single-platelet adhesion and small aggregates observed in the lungs, but no large platelet aggregates or emboli (Figure 3A). Importantly, both doses restored lung perfusion, suggesting that neutrophil recruitment and adhesion, along with minimal platelet adhesion and fibrin deposition, contribute to the hypoperfusion in the pulmonary microcirculation.

### Syk Inhibition Reduced Platelet and Neutrophil Recruitment and Hypoperfusion in Organ-Specific Manner

**Figure 4.**
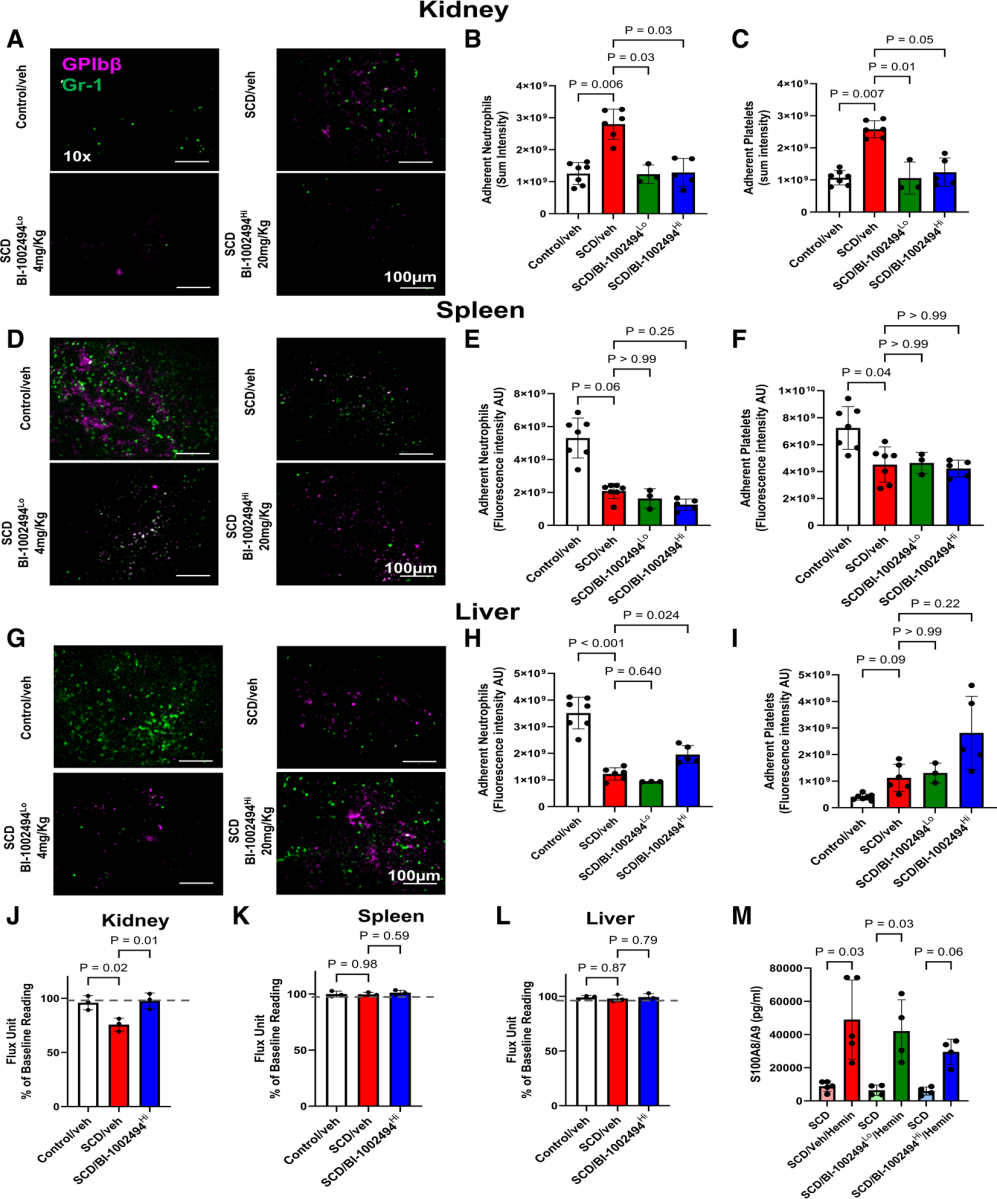
**Extracellular hemin alters neutrophil and platelet recruitment and blood perfusion in an organ-specific manner.** Hemin (20 µmol/kg, IV) was injected into control and sickle cell disease (SCD) mice. Neutrophil and platelet recruitment to the kidney, spleen, and liver was analyzed at 60 minutes posthemin and imaged at ×10 magnification. **A**, Representative intravital images of the kidney showing adherent neutrophils (Gr [granulocyte-receptor antigen]-1; green) and platelets (GP [glycoprotein] Ibβ; magenta) in the renal microcirculation. Quantitative analysis of the intravital data for fluorescence intensity arbitrary unit (AU; **B**) total adherent neutrophils, and (**C**) platelets. **D**, Representative intravital images of splenic microcirculation showing adherent neutrophils and platelets. Quantitative analysis of the intravital data for fluorescence intensity AU for (**E**) total adherent neutrophils and (**F**) platelets. **G**, Representative intravital images of the liver showing adherent neutrophils and platelets in the hepatic microcirculation. Quantitative analysis of the intravital data for fluorescence intensity AU for (**H**) total adherent neutrophils and (**I**) platelets (n=3–7 mice). Quantitative analysis of flux unit readings as a percentage of baseline values obtained by laser speckle contrast imaging (LSCI) for the (**J**) kidney, (**K**) spleen, and (**L**) liver (n=3 mice). **M**, Plasma level of S100A8/A9 was measured by ELISA in SCD mice before and 40 minutes after hemin injection. The statistical significance between multiple groups was analyzed using the Kruskal-Wallis test with multiple comparisons compared with the SCD/veh group for **B**, **C**, **E**, **F**, **H**, **I**, and **M** with Dunn multiple comparisons. For **M**, a comparison between SCD before hemin and the same group after hemin was performed. An ordinary 1-way ANOVA with Dunnett multiple comparisons was performed for **J** through **L**.

In an experimental model of SCD, hemin injection led to its accumulation in the kidney and triggered acute kidney injury, whereas in control mice, hemin accumulated primarily in the liver.^47^ We next evaluated whether hemin induced systemic alterations in cell recruitment and organ hypoperfusion. These measurements were performed in parallel with lung intravital imaging, comparing platelet and neutrophil recruitment in the kidney, liver, and spleen at baseline and 1-hour posthemin injection. At baseline, neutrophil and platelet retention was higher in the kidney of SCD mice compared with controls. Intravital imaging revealed that hemin injection significantly increased platelet and neutrophil recruitment to the kidney in SCD mice, an effect that was reduced by both doses of BI-1002494 (Figure 4A through 4C). In contrast, neutrophil recruitment to the liver and spleen was reduced in SCD mice compared with controls, with BI-1002494 treatment having no effect on recruitment to these organs (Figure 4D, 4E, 4G, and 4H). Notably, the impaired neutrophil recruitment to the liver in SCD mice was partially reversed by the higher dose of BI-1002494 (20 mg/kg), but not the lower dose (4 mg/kg; Figure 4G and 4H). Platelet recruitment to the spleen was also reduced in SCD mice without further change following BI-1002494 treatment (Figure 4F), and no significant difference was observed in platelet recruitment to the liver between SCD and control or BI-1002494-treated mice (Figure 4I). Reduced neutrophil and platelet recruitment to the kidney was associated with improved renal perfusion, although perfusion in the spleen and liver remained unchanged (Figure 4J through 4L), with both organs showing lower perfusion at baseline in SCD mice compared with controls (Figure S2G and S2H). The increase in S100A8/A9 levels following hemin injection was unaffected by the low dose of BI-1002494 (4 mg/kg), whereas the higher dose (20 mg/kg) slightly reduced S100A8/A9 levels compared with vehicle (Figure 4M), suggesting that BI-1002494 primarily impacts neutrophil adhesion in this experimental model. These findings highlight organ-specific changes in neutrophil and platelet recruitment following hemin challenge in SCD mice, linked to impaired perfusion, and demonstrate that BI-1002494 improves renal perfusion following hemin administration.

### Syk Activation Contributed to Inflammatory Bleeding in Hemin-Treated SCD Mice

**Figure 5.**
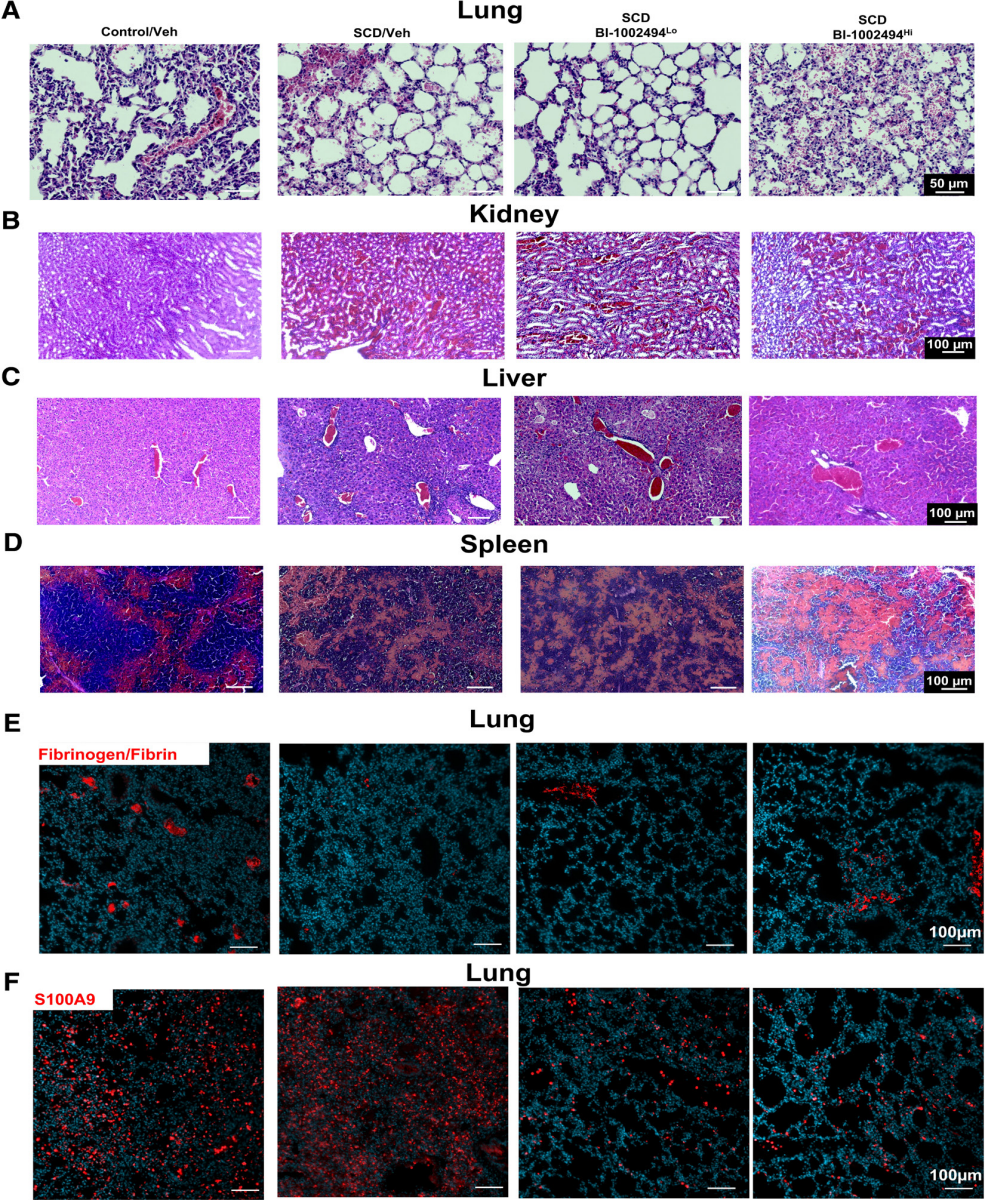
**Low-dose BI-1002494 reduced hemin-induced bleeding in the lung.** BI-1002494 (4 or 20 mg/kg) or vehicle (veh) was injected in ventilated sickle cell disease (SCD) mice via intraperitoneal route 30 minutes before the injection of hemin (20 µmol/kg, IV). Lung, kidney, liver, and spleen were fixed for 60 minutes post-hemin injection. Formalin-fixed paraffin-embedded (FFPE) sections were stained using (**A** through **D**). **E** and **F**, Immunofluorescence staining for (**E**) fibrinogen/fibrin and (**F**) S100A9 in FFPE sections. Nuclei are stained with Hoechst 33342 (blue). Scale bar 50 µm for (**A**) and scale bar 100 µm for (**B** through **F**). H&E (hematoxylin and eosin) and immunofluorescence staining were imaged using a Zeiss Axio Scan microscope and analyzed using Zen software (representative image of 3 mice per group).

Consistent with previous reports,^19^ we observed that hemin induced bleeding in the lungs of SCD mice (Figure 1C and 5A). Hemin-induced lung bleeding was alleviated by low doses of BI-1002494, but exacerbated by higher doses (Figure 5A). In contrast to the lungs, red cell congestion was observed in other organs of hemin-treated mice, and this congestion persisted in BI-1002494-treated mice (Figure 5B through 5D). BI-1002494 had minimal impact on fibrinogen/fibrin deposition in the lungs (Figure 5E), but a reduction in S100A9-positive cells was noted in BI-1002494-treated mice, with no evidence of S100A9 deposition on the vessel wall (Figure 5E). The decrease in S100A9-positive cells, predominantly neutrophils, confirmed that neutrophil recruitment to the lung microcirculation is Syk-dependent. These findings suggest that targeting Syk may be a promising strategy to limit neutrophil recruitment and improve lung and kidney perfusion following hemin challenge.

## Discussion

This study used intravital imaging and laser speckle contrast imaging to examine organ-specific effects of hemin on neutrophil and platelet recruitment and blood perfusion in SCD mice, while evaluating the impact of Syk inhibition on vaso-occlusion. Hemin increased neutrophil and platelet recruitment to the ventilated lung, impairing perfusion independently of fibrin generation and RBC sickling, while causing sickling and congestion in the kidney, liver, and spleen. Syk inhibition with BI-1002494 reduced neutrophil and platelet adhesion in the lung and kidney, improving perfusion. Both doses of BI-1002494 inhibited neutrophil recruitment and large platelet aggregates in the lung; however, the low dose reduced pulmonary bleeding, whereas the high dose exacerbated it.

VOC can be induced by factors, such as physiological stress,^48^ cold temperature,^49^ hemolysis,^50^ hypoxemia, and heightened inflammatory states. Among these, hemolysis stands out as a key driver, releasing heme, which is quickly oxidized into hemin and drives VOC and ACS in different humanized SCD mice (Berkeley, Townes).^18,19^ Our intravital imaging uncovered organ-specific recruitment of neutrophils and platelets in response to hemin, with marked perfusion impairments in the lung and kidney but not in the spleen and liver. The latter organs displayed baseline hypoperfusion due to chronic inflammation, hemolysis, and excessive RBC clearance. Notably, the decrease in pulmonary perfusion was linked to increased recruitment of S100A9-positive neutrophils, highlighting that hemin fosters neutrophil adhesion. In human cell studies, we observed that hemin enhanced neutrophil adhesion to VWF and fibrinogen in a Syk-dependent manner. PSGL-1 mediates transient adhesion by directly interacting with VWF, whereas neutrophil activation leads to firm adhesion through β2 integrins, which bind to poly-RGD peptides,^27,29,51^ suggesting that the RGD sequence of VWF and fibrinogen could be involved in adhesion.

Hemin-induced platelet activation also triggered NETosis in a Syk-dependent manner, confirming that both platelet and neutrophil Syk contribute to hemin-induced NETosis. This process might involve key players like P-selectin and HMGB (high-mobility group box)-1, which are essential for activated platelet-induced NETosis. Although blocking P-selectin resolves pulmonary arteriole microemboli,^8^ thrombo-inflammation, and embolic NETs persisted in LPS-challenged SCD mice.^18,52^ Additionally, hemin-activated platelets induced NETosis via a PKC-MEK-ERK-dependent mechanism, with ferroptosis and iron chelation suppressing this process.^23^

Beyond NETosis, hemin-activated platelets also promoted the release of S100A8/A9 from neutrophils, amplifying neutrophil recruitment, NETosis, and platelet activation. The effect of BI-1002494 may extend beyond neutrophils and platelets to influence endothelial activation by hemin. Indeed, hemin induces endothelial activation via TLR-4, and Syk plays a crucial role in TLR-4 downstream signaling.^18^ We have previously shown that hemolysis-induced platelet recruitment to endothelial cells requires endothelial cell activation, partially mediated by the expression of VWF.^53^ Interestingly, hemin injection did not increase plasma VWF levels, likely because VWF is retained on the surface of endothelial cells, potentially exacerbating platelet activation and neutrophil adhesion. Interestingly, BI-1002494 did not significantly reduce S100A8/A9 release despite a slight decrease observed with the high dose of BI-1002494. It is possible that a combination of therapies may be required to block neutrophil activation and adhesion across multiple organs.

In addition to neutrophil and platelet recruitment, hemin induced bleeding in the lungs of SCD mice, likely due to a combination of inflammatory bleeding and altered endothelial permeability.^26,54,55^ Inflammatory bleeding is mediated by neutrophil adhesion and transmigration,^56^ features commonly observed in SCD. The contribution of platelet receptors to the maintenance of vascular integrity is influenced by specific organs and stimuli^54,57^; platelet dysfunction and thrombocytopenia in SCD mice may compromise these protective functions. Notably, baseline and agonist-induced platelet activation are elevated in Berkeley mice, a priming phenotype increasing with age.^58^ We observed a dose-dependent effect of BI-1002494 on bleeding, with lower doses reducing bleeding, whereas higher doses exacerbated it. The bleeding observed at high doses of Syk inhibitor is unlikely to be simply an exacerbation of inflammatory bleeding; it may result from hemin-induced endothelial cell death, which increases vascular permeability.^59^ This is supported by findings showing reduced neutrophil adhesion and transmigration in mice treated with high doses of BI-1002494. These results, along with data from low-dose BI-1002494–treated mice, suggest that platelet adhesion, without large aggregate formation, may help preserve vascular integrity during vaso-occlusive events. The safety of Syk inhibitors administered to patients or currently evaluated in clinical trials^36,37,60^ indicates that high doses of BI-1002494 may be the issue. The identification of neutrophil adhesion in the pulmonary microcirculation as the major driver of hypoperfusion suggests that Syk inhibitors with low effects on platelets and thrombosis in vivo, such as R406 (the active metabolite of Fostamatinib),^61^ could be effective and safe to improve ACS in SCD; however, their efficacy remains to be determined.

This study has several limitations that should be acknowledged. First, intravital imaging was performed exclusively on the left lobe of the lung, leaving uncertainty as to whether the observed cellular dynamics are consistent across all lobes. Although histological analysis of the right lobes and studies involving SCD mice^9^ suggest that these changes are not restricted to a specific lobe, the generalizability of our findings remains uncertain. In this study, cell recruitment was analyzed by intravital imaging of the left lung lobe, while visualizing deeper lobes, such as the right caudal and accessory lobes, was challenging using this cellular-level imaging technique. Recent advancements in intravital imaging have enabled visualization of the right lobes.^62^ Future studies, that directly compare cellular dynamics across different lobes, could provide a more comprehensive understanding of potential inter-lobar differences. This line of investigation is particularly important because ACS in patients with SCD predominantly affects the lower lobes in adult patients, whereas the upper and middle lobes are more frequently involved in children.^63^ ACS is attributed to 3 primary causes: infection, fat embolization from damaged bone marrow, and pulmonary infarction. Notably, infection is the most common cause of ACS in pediatric patients. Future research exploring the impact of various stimuli on ACS in different lobes could offer critical insights into the underlying mechanisms driving lobe-specific manifestations and responses.

Another limitation pertains to the specificity of BI-1002494, which was not explicitly validated in vivo. Although previous studies have established BI-1002494 as a selective Syk inhibitor for both in vitro and in vivo applications,^46,64^ the possibility of off-target effects on other kinases cannot be entirely excluded. Indeed, our data show that high doses of BI-1002494 can affect Src phosphorylation, which may potentiate bleeding side effects. As BI-1002494 was tested in vivo in Berkeley mice, a dose-response study in different models might help determine whether these effects are specific to SCD.

In conclusion, our study demonstrates that hemin-challenged SCD mice exhibit organ-specific mechanisms, with monitoring cell recruitment and perfusion providing insight into the efficacy of Syk inhibition in reducing platelet aggregates and neutrophil recruitment in the lung and kidney. This work offers in vivo proof-of-concept for repurposing Syk inhibitors to treat ACS in SCD. Combining low-dose Syk inhibitors with therapies aimed at reducing RBC sickling may provide enhanced protection against VOC and ACS. However, the long-term effectiveness of Syk inhibitors in preventing progressive organ decline in SCD remains to be evaluated, particularly considering the potential of heme to induce trained immunity via Syk, which may influence the response to infections.^65^

## Article Information

### Acknowledgments

J. El-Awaisi performed intravital and laser speckle experiments, collected, analyzed data, and contributed to data interpretation; G. Perrella, N. Mayor, V. Tinkova, and B. Grygielska performed experiments, collected, analyzed data, and prepared figures; S.J. Cleary developed methodology for intravital imaging; S.P. Watson, A. Brill, J.D. Dimitrov, E. Hassan, and P.L.R. Nicolson contributed to data interpretation; D. Kavanagh and N. Kalia provided key technical expertise and support for intravital imaging and laser speckle contrast imaging and analysis; J. Rayes designed research and experiments, performed experiments, analyzed data, prepared figures, and wrote the article; all authors read and approved the article. BI (Boehringer Ingelheim) 1002494 was kindly provided by Boehringer Ingelheim via its open innovation platform opnMe, available at https://www.opnme.com. The authors would like to thank the Biomedical Services Unit at the University of Birmingham for their support and expertise when undertaking the in vivo aspects of this work. The authors acknowledge the support of the University of Birmingham– Microscopy Facility, RRID:SCR_027108, and the intravital imaging facility, RRID:SCR_027189, for providing access to equipment and technical expertise. To comply with open-access journal subject CC-BY licensing, the cover figure and Figure S1A made using biorender.com will be made publicly available on publication.

### Sources of Funding

J. Rayes holds a British Heart Foundation (BHF) Intermediate Fellowship (FS/IBSRF/20/25039). G. Perrella was supported by a BHF Project grant awarded to J. Rayes (PG/21/10737). V. Tinkova was supported by a BHF studentship (FS/PhD/23/29422). J. El-Awaisi was supported by a BHF Project grant awarded to N. Kalia (PG/21/10574). A. Brill holds a BHF Senior Fellowship (FS/19/30/34173). The National Institutes of Health and Care Research (NIHR) Birmingham Biomedical Research Center (NIHR203326) and the BHF Accelerator (AA/18/2/34218) have supported the University of Birmingham Institute of Cardiovascular Sciences, where this research is based. The opinions expressed in this article are those of the authors and do not represent any of the listed organizations.

### Disclosures

P.L.R. Nicolson has received a research grant from Rigel. The other authors report no conflicts.

### Supplemental Material

Figures S1–S6

Videos S1–S3

Major Resources Table

ARRIVE Guidelines

Full Unedited Gels for Western Blots
